# Effects of tai chi program on neuromuscular function for patients with knee osteoarthritis: study protocol for a randomized controlled trial

**DOI:** 10.1186/1745-6215-14-375

**Published:** 2013-11-07

**Authors:** Xue-Qiang Wang, Ling-Yan Huang, Yu Liu, Jing-Xian Li, Xie Wu, Hai-Peng Li, Lin Wang

**Affiliations:** 1 Key Laboratory of Exercise and Health Sciences, Ministry of Education, Shanghai University of Sport, Shanghai 200438, China; 2 School of Human Kinetics, Faculty of Health Sciences, University of Ottawa, Ottawa, Canada

**Keywords:** Knee osteoarthritis, Neuromuscular function, Physical therapy, Randomized controlled trial, Tai chi

## Abstract

**Background:**

Knee osteoarthritis (OA) is a major cause of disability as well as a burden on healthcare resources. Tai chi has been proposed as an alternative and complementary treatment for the management of knee osteoarthritis, but there appears to be no consensus on its usefulness. This study aims to develop an innovative tai chi rehabilitation program (ITCRP) for patients with knee OA, and to investigate the effect of ITCRP intervention on a range of outcomes including pain, function, balance, neuromuscular response, and biomechanics in knee OA.

**Methods/Design:**

We will conduct a prospective, single-blind, randomized controlled trial of 140 individuals with symptomatic knee OA. Patients will be randomly assigned into either an ITCRP group or a control group. The ITCRP group will participate in tai chi two or three times a week for 6 months. The control group will receive health education. After the 6-month intervention period, there will be a 6-month follow-up period with no active intervention in either group. The primary and secondary outcomes will be assessed at baseline, 6 months, and 12 months. Primary outcome measures will be a visual analog scale for pain, the Western Ontario and McMaster Universities Osteoarthritis Index,and the Lequesne Knee Score. The secondary outcome measures will include the Berg balance scale, knee and ankle proprioception, neuromuscular response, and 3D functional biomechanics. Furthermore, adverse events will be recorded and analyzed. If any participants withdraw from the trial, intention-to-treat analysiswill be performed.

**Discussion:**

Important features of this trial include the randomization procedures, large sample size, and a standardized protocol for ITCRP for knee OA. This study aims to determine the feasibility of ITCRP for knee OA and provide data on the effects of ITCRP. Hence, our results will be useful for patients with knee OA as well as for medical staff and healthcare decision makers.

**Trial registration:**

Chinese Clinical Trial Registry: ChiCTR-TRC-13003264.

## Background

Knee osteoarthritis (OA) is a major cause of disability, especially for older people, as well as a burden on healthcare resources [1,2]. Age is the strongest predictor of knee OA and therefore increasing age and extended life expectancy will result in a greater occurrence of the disease [3]. The impact of OA can be severe and profound, and OA often results in both direct and indirect medical expenses. In the United States, the total cost of arthritis and other rheumatic conditions in 1997 was $116.3 billion, comprising direct costs of $51.1 billion (hospitals, doctors, transportation, nursing homes, and so on) and indirect costs of $65.2 billion (primarily lost wages and lost productivity) [4].

Patients with knee OA experience pain and loss of function. At present, there is no cure for knee OA. The management of knee OA is broadly divided into nonpharmacological, pharmacological, and surgical treatments [5]. Surgical treatments are often recommended if other treatments are ineffective and functional disability affects the patient’s quality of life. Pharmacological management includes control of pain and improvement in function and quality of life. However, pharmacological agents used to treat the symptoms of knee OA are associated with various sideeffects [6]. Exercise is recommended for the nonpharmacological management of knee OA [7], but many forms of exercise may betoo intense, uncomfortable, or monotonous for older adults to maintain over an extended period of time [8]. In 2012, the American College of Rheumatology conditionally recommended tai chi for patients with knee OA [9]. Tai chi is a popular form of exercise among older adults, especially in Asia; it encompasses balance, aerobics, flexibility, and weight-bearing exercise with meditation and deep breathing. Tai chi involves a series of slow, smooth, and graceful movements, with an emphasis on smooth coordination of the eyes, head, body, and upper and lower extremities [8].

There are five primary types of tai chi (Yang style, Wu style, Chen style, Hao style, and Sun style), and each style takes a different approach in terms of the movements and forms. For every type of tai chi, there are many forms, such as the 24-, 36-, and 48- form Yang styles. The biomechanical characteristics, in terms of joint loading, muscle activity, and range of lower limb motion, of the tai chi movements in the intervention programs from the published studies have not been investigated. Without an understanding of tai chi biomechanics, the mechanisms of the effects of tai chi intervention for OA management would be unknown. Recently our research team has studied the biomechanical characteristics of some of the most representative tai chi movements [10,11]. The understanding of the biomechanics of these tai chi movements gained from our studies and other published work [12] provides the scientific basis for developing an innovative tai chi rehabilitation program (ITCRP) specifically for OA patients.

Most previous studies [13,14] of regular tai chi programs only focus on pain relief and maintenance of the range of motionof the joint for knee OA, rarely considering the importance of neuromuscular control. Thus, it is not known whether exercise interventions influence factors associated with progression of OA. This study will not only examine the effects of a tai chi intervention on knee pain andrange of motion, but will also focus on joint biomechanics, muscle strength, proprioception tests of knee and ankle, and neuromuscular response. Hence, we aim to investigate the efficacy of a 6-month ITCRP compared with a 6-month health education program on a broad range of outcomes in patients with knee OA.

## Methods/Design

### Study design

**Figure 1:**
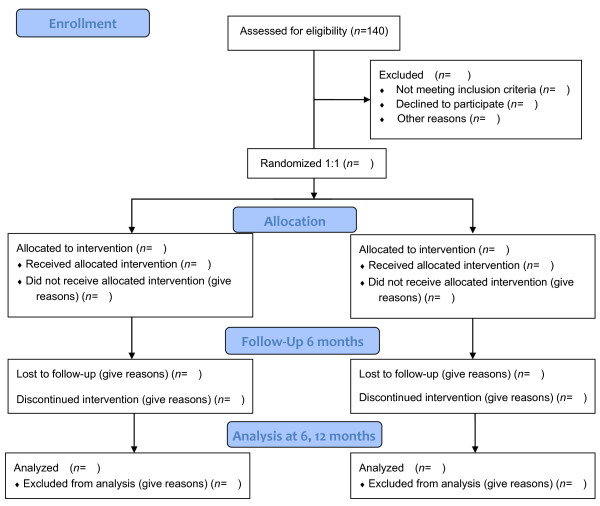
Flow diagram of study design.

This is a single-blind randomized controlled trial comparing a tai chi program with health education (Figure 1). The investigative sitesare the Shanghai University of Sport and the Shanghai Shangti Orthopedic Hospital. A total of 140 patients will be included from Guohe community center, Lanxin community center, and Dongfang community center, all in Yangpu District, Shanghai City, China.

Prior to initiation of the study, all subjects will complete a questionnaire asking for details including medical history and past and present job status. Subjects will also complete the Mini–Mental State examination and the Activity of Daily Living test, and will describe their exercise habits (frequency and duration). Informed consent will be sought from all subjects prior to inclusion in the study.

The participants who meet the inclusion criteria will be allocated in a 1:1 ratio by computer-generated randomization. After randomization, participants will be assigned to either the tai chi group or the health education group (control). The study will include assessments at the following time points: before intervention, after an intervention period of 6 months, and after a further follow-up period of 6 months with no active intervention. The total study period will be 12 months.

### Participants

#### Inclusion criteria

Participant selection will be based on the Classification Criteria of the American Rheumatism Association for knee OA [15].

1. Men or women, with diagnostic criteria of definite OA of the knee (unilateral or bilateral) according to radiography with reports of pain symptoms for at least 3 months;

2. Mild to moderate knee OA (Lequesne Knee Score = 1 to 7);

3. Age 60 to 90 years;

4. Medication not expected to change during the study period;

5. Availability: three times a week over a period of 12 months.

#### Exclusion criteria

Participants with any of the following conditions will be excluded:

1. Inability to communicate in English, French or Chinese;

2. Presence of pain at rest or at night;

3. A medical condition involving hip or knee trauma, or intra-articular hip, or knee injection within one month;

4. Exercise-induced or uncontrolled angina within three months, or severe dyspnea at rest;

5. Terminal illness;

6. Uncontrolled hypertension;

7. Other illness, judged by the patient or study physician to make participation in this study inadvisable;

8. Bilateral total hip or knee arthroplasties;

9. Current rehabilitation treatment or previous corticosteroids injection within the last 12 months or any other pain-related treatment besides medication for arthritis;

10. A Mini-Mental State Exam score of 23 or lower [16];

11. Surgery planned in the next year;

12. Obesity (body mass index >30 kg/m^2^).

### Withdrawal criteria and management

Study patients will be allowed or asked to withdraw from the study if:

1. The participant or his or herlegal representative makes such a request.

2. The participant develops a serious disease, such as heart disease or stroke and, in the investigator’s opinion, it is not suitable to continue.

3. The participant has an adverse reaction related to the tai chi exercise program.

### Recruitment of patients

People with mild to moderate hip or knee OA will be recruited through local television, newspaper advertisements, and flyers.

### Ethical considerations

The study was approved by the ethics committee of the Shanghai University of Sport, China. Before the randomization, all patients will be requested to sign a written informed consent statement.

### Interventions

#### ITCRP group

**Figure 2:**
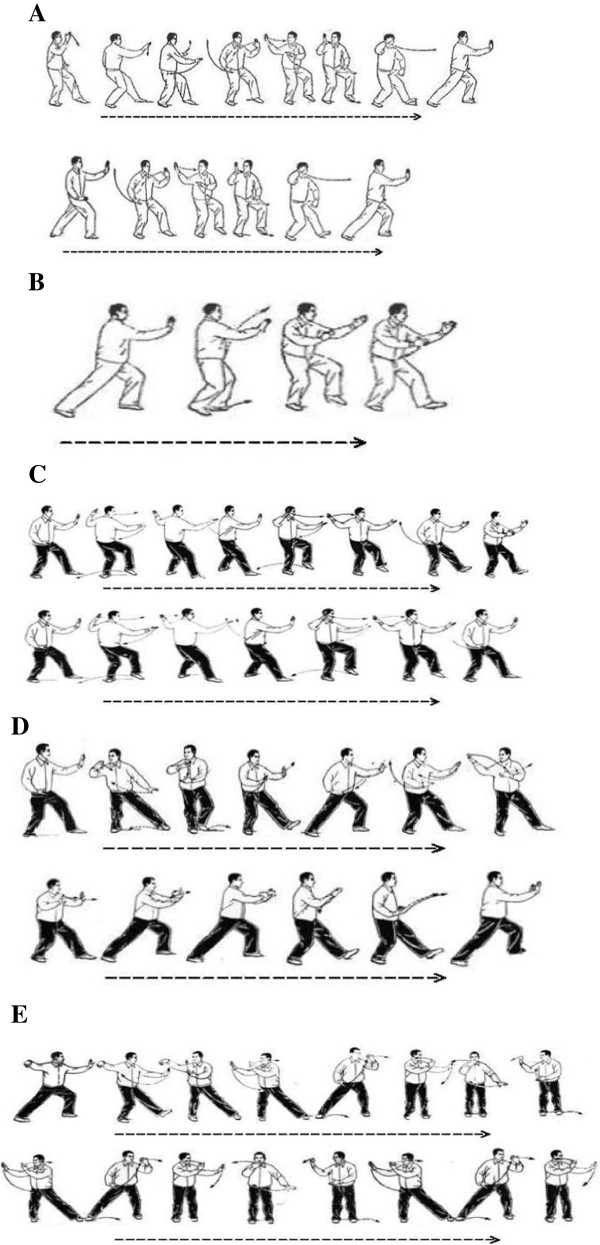
**Illustration of five tai chi movements. (A)** Brush Knee and Twist Steps; **(B)** Playing the Lute; **(C)** Step Back to Repulse Monkey; **(D)** Grasp Sparrow’s Tail; **(E)** Wave Hands like Clouds.

The details of the ITCRP were developed specifically for OA patients by an expert panel comprising a qualified tai chi instructor and others in the research team using a collaborative, iterative process known as the Delphi method [17]. The group used expert knowledge of tai chi practice, knee OA, and the biomechanical characteristics of tai chi movements as analyzed by our team [10,11,18] and other biomechanics researchers [12]. One of the strengths of ITCRP is the application of user-friendly, easily-learnt movements. The five chosen tai chi movements in ITCRP are shown in Figure 2.

In the first four weeks of the ITCRP intervention, the participants will be taught tai chi for two sessions per week. Each session will last an hour and will be supervised by a tai chi instructor in a community center. Each session will include a warm up for 10 minutes, learning new movements for 20 minutes, reviewing the learnt movements for 20 minutes, and cooling down for 10 minutes. Participants will be provided with the tai chi practice manual. In subsequent weeks, participants will practice tai chi individually or in groups for three sessions per week, with each session lasting one hour. Each session will include a 10-minute warm up, a 45-minute practice and refinement, and a 5-minute cool down. The instructor will visit each group or individual weekly to ensure that the movements are being correctly practiced.

The participants will be asked to fill out forms to record the times and durations of tai chi practice. The forms will be returned to the researchers for monitoring in a weekly meeting. The choice of 6 months as the intervention period is based on the published literature on tai chi intervention studies for knee OA patients [19-21].

After a 6-month ITCRP intervention, there will be a 6-month follow-up period with no active intervention. During the follow-up period, the participants will be asked to fill out forms to record the times and durations of tai chi or other physical activity. The forms will be returned to the researchers for following up each week by email or mail. A monthly gathering will be organized, for discussion of health-related topics and detailed follow-up.

#### Control group

The participants in the control group will attend one 60-minute group session per week. The session will consist of a 30-minute lecture, followed by a 30-minute discussion. The lectures will cover health-related topics, such as OA, aging, nutrition. The participants in the control group will be asked to maintain their previous lifestyle and not take part in any other regular rehabilitation programs.

### Outcome measures

All outcome measures will be administered by the main research assistant, blind to group allocation, at 2 weeks (baseline), 6 months (follow-up 1, at the end of intervention) and 12 months (follow-up 2). Demographic information will include age, sex, ethnicity, marital status, educational status, accommodation type and postcode. Clinical information will also be obtained from the patients’ clinical records by a member of their hospital research team.

#### Primary outcome measures

1. Visual analog scalefor pain [22]. This uses a horizontal line, 100 mm in length, marked on the left as 'no pain’ (score 0) and on the right as'unbearable pain’(score 10). The patient marks on the line the point representing their perception of their current state. The amount of pain that a patient feels ranges across a continuum from none to an extreme amount of pain.

2. Western Ontario and McMaster Universities Osteoarthritis Index [23]. This is a widely used, proprietary set of standardized questionnaires used by health professionals to evaluate the condition of patients with osteoarthritis of the knee and hip, including pain, stiffness, and physical functioning of the joints. The indexmeasures five items for pain (score range 0 to 20), two for stiffness (score range 0 to 8), and 17 for physical function (score range 0 to 68).

3. Lequesne Knee Score [24]. This can be used to assess the effectiveness of therapeutic interventions for osteoarthritis for the knee. Total score ranges from 0 to 24, with lower scores indicating less functional impairment. The classification is as follows: >13,extremely severe;11 to 13, very severe; 8 to 10, severe; 5 to 7, moderate; 1 to 4,mild; 0, none.

#### Secondary outcome measures

1. Berg balance scale [25]. This is a widely used clinical test of a person’s static and dynamic balance abilities, which comprises a set of 14 simple balance related tasks, ranging from standing up from a sitting position, to standing on one foot. Total score ranges from 0 to 56, with 0 to 20 corresponding to a high fall risk, 21 to 40 a medium fall risk, and 41 to 56 a low fall risk.

2. Time up and gotest [26]. This reflects the time that a person takes to rise from a chair, walk three meters, turn around, walk back to the chair, and sit down. Times up to 10 secondsindicate normal mobility, 11 to20 seconds are within normal limits for frail elderly and disabled patients, and times longerthan 20 seconds signify thatthe person needs assistance and indicates further examination and intervention.

3. Functional reach test [27]. This is a dynamic rather than a static test and measures a person’s 'margin of stability’ as well as the ability to maintain balance during a functional task. The distance between the start and end points is measured using the head of the metacarpal of the third finger as the reference point. Three trials are done and the average of the last two is noted.

4. Muscle strength test of knee and ankle. The muscle strength on the dominantknee and ankle joint will be tested using an isokinetic dynamometer (850–000, Biodex, New York, USA). Knee muscle strength and endurance will be determined using the method used in our previous tai chi intervention studies [18,28]. Participants will perform three maximum concentric contractions for knee extensors and flexors at the angular velocities of 30°/s. The highest peak torque indicating muscle strength will be normalized by kilogram of body weight. The dynamic endurance of the knee extensors and flexors will be assessed by measuring 40 repeated maximum isokinetic contractions with an angular velocity of 180°/s. The work from a knee angle of 80° to 10° will be recorded for each contraction. The endurance index is defined as the ratio of the work done during the last five contractions over the first five contractions. Ankle muscle strength will be measured as in our previous studies [18,28]. Ankle dorsiflexor and plantarflexor strength will be measured at an angular velocity of 30°/s. Participants will be instructed to push afoot away from themselves and then pull it toward themselves at the maximum velocity for each action. Peak torque will be determined as the highest torque generated from the three trials. In addition, the relative peak torque will be analyzed.

5. Proprioception test of knee and ankle. The measurement method has been reported in our previous studies [29]. Knee and ankle proprioception will be tested using an electrically driven movable frame. During the tests, the participants will sit on a chair with their dominant legs supported by the frame. The leg can be passively moved in a flexed or extended direction at a velocity of 0.4°/s. Once the participant is able to detect the motion of the leg, he or she will press a handheld stop button and confirm the direction of the motion. The rotation angles of the frame will be determined as the threshold for the detection of the knee and ankle joint. The mean values of the three trials in one direction will be used for the analysis.

6. Neuromuscular response. Neuromuscular response indicated by muscle latency will be assessed through the use of electromyography (EMG) of the leg muscles, while an unexpected perturbation is applied to the ankle. We have used the method in our previous study [18]. A customized trap door with an 18° tilt angle will be used to generate an ankle inversion perturbation while subjects stand barefoot on trap doors. The Bagnoli-8 EMG system (DS-B04, Bagnoli™-16 Desktop EMG system, Delsys Inc., Boston, MA) will be used to collect surface EMG signals in four muscles (rectus femoris, semitendinosus, gastrocnemius, and anterior tibialis) of the right leg and the onset signals at the trap door tilting. To reduce anticipatory effects, both feet will be randomly tilted at least seven times. The onset latency of the muscles refers to the time interval in milliseconds (ms) between trapdoor initiation and the first rising front of the EMG burst from the baseline. The EMG onset will be determined visually by an experienced researcher.

7. Three-dimensionalfunctional biomechanics. The importance of the mechanical component in the progression of knee OA is evident. Biomechanical analysis provides a noninvasive and reproducible assessment of dynamic loading on the joints during movement. A three-dimensionalbiomechanical analysis is necessary to determine the mechanism by which kinematics and kinetics of the joints change during intervention and provide a quantitative analysis of the effect of the intervention [30,31]. Kinematic data will be acquired with a three-dimensionalmotion capture system (Vicon, Oxford, UK) and, in combination with ground reaction force data and EMG data, will be used to derive three-dimensionalkinematics and kinetics variables. These biomechanical data will be collected while each participant performs four tasks: (1) level-walking at a self-determined natural speed; (2) sitting from a standing position and standing from a seated position; (3) ascending and descending a three-step staircase; and (4) ascending and descending a ramp with a 12.5% slope as per the Ontario Building Code Act. For data collection of joint kinematics and EMG of lower limb, instrumentation, and data collection protocols will be the same. For joint kinetics analysis, ground reaction force will be recorded. Four force platforms (9286AA, Kistler Instruments Corp, Winterhur, Switzerland; FP 4060–08, Bertec Corporation, Columbus, OH, USA) are embedded in the middle of the walkway orin the first and second steps of the staircase. Participants will be asked to walk, or ascend and descend at their natural speed without any reference to the force platforms. In chair rising and sitting, the participants will be instructed to use their lower extremities only, without any help from their arms. The chair is 45 cm high and 43 cm deep.

8. Pittsburgh Sleep Quality Index [32]. This is a self-rated questionnaire, which assesses sleep quality and disturbances. It contains 19 questions answered by the subject themselves and 5 questions answered by the bed partner or roommate (if one is available). The scores from seven domains are added to calculate the index, which ranges from 0 to 21. A score of zero indicatesno disturbance in sleep or good sleep quality, whereas higher scores indicate poorer sleep quality.

9. Activities of daily living scale [33]. We will use the Barthel Index, which consists of tenitems to measure a person’s daily functioning, specifically the activities of daily living and mobility. The items include feeding, moving from wheelchair to bed and return, grooming, transferring to and from a toilet, bathing, walking on level surface, going up and down stairs, dressing, continence of bowels and bladder. Total scores are calculated by summing the individual item scores. Scores are weighted and range from 0 (dependence) to 100 (independence). Scores of 0 to 20 indicate total dependence; 21 to 60, severe dependence; 61 to 90, moderate dependence and 91 to 99, slight dependence.

10. Short Physical Performance Battery [34]. This is a tool designed to quantify physical performance and decline over time. The test focuses primarily on lower extremity function and includes a 4-meter walk to measure gait speed, one chair stand (followed by five timed chair stands, if the first is successfully completed), and balance stands with the feet in different positions for 10 seconds each. Scores are weighted and range from 0 (worst performance) to 12 (best performance). Scores of 0 to 3 indicate severe limitations; 4 to 6, moderate limitations; 7 to 9, mild limitations and 10 to 12, minimal limitations.

11. SF-36 Health Survey [35]. This is a multi-purpose, short-form health survey with only 36 questions. SF-36 items cover eight domains: physical functioning, role limitations due to physical health problems, bodily pain, general health, vitality, social functioning, role limitations due to emotional problems and mental health. Higher scores indicate higher levels of health.

12. Adverse events. A record will be made of any side effects and possible adverse reactions arising from the ITCRP.

### Statistical analysis

Statistical analyses will be performed using SPSS 17.0 and Microsoft Excel 2007 software. Data will be expressed as mean ± standard deviation. Changes in variables before and after training and between groups will be analyzed. The main analysis will be to compare the efficacy of ITCRP group with the control group, including the primary and secondary outcomes, using 2-way analysis of variance (group × time). We will conduct an intention-to-treatanalysis if participants are lost to follow-up. The basic intention-to-treat principle is that participants in trials should be analyzed in the groups to which they were randomized, regardless of whether they received or adhered to the allocated intervention. Statistical significance was assumed at *P* less than 0.05.

## Discussion

Adequate and appropriate mechanical loading is an essential stimulus to maintain physiological joint homeostasis, whereas excessive mechanical stress at the joint triggers the onset and progression of knee OA. Based on joint, tissue, and movement biomechanics, tai chi is an ideal approach in the management of lower limb OA because tai chi movements are characterized by low impact, low loading rate, an accompanying neuromuscular training effect, and a specific gentle gait pattern [36]. The highest knee joint reaction forces when performing tai chi movements are equivalent to only 1.2 times body weight [12], significantly less than the joint reaction force when walking (3 times body weight) [37]. The special tai chi stepping pattern, characterized by multidirectional foot movements with gentle foot landings, may provide good gait training for patients with knee OA [31,38]. Tai chi is also a low-cost exercise that can be performed at home without the use of any special equipment.

However, findings of studies on tai chi for OA are very difficult to compare because tai chi intervention programs differ in terms of duration, style, and movements. Some tai chi styles and movements are difficult for older people to learn, and not all tai chi styles and movements are suitable for knee OA.

### Strengths and limitations

Firstly, the proposed research program is unique in that the ITCRP intervention is specifically designed for subjects with knee OA. Secondly, the study duration is relatively long, with an intervention period of 6 months and a follow-up period (with no active intervention) of 6 months, giving a total study period of 12 months. Previous tai chi intervention studies have typically ranged in duration from a few weeks to six months [39]. Thirdly, we will use a full three-dimensional biomechanical analysis to determine the mechanism by which kinematics and kinetics of the joints change during intervention and provide a quantitative analysis of the effect of intervention. The impact of tai chi on joint biomechanics remains unclear. Previous studies have focused on knee range of motion, knee pain, joint stiffness, walking speed, and stride length, but three-dimensional joint biomechanics of patients with OA has not yet been analyzed. Published studies have suggested that gait assessment without full three-dimensional analysis provides only limited information on the level of joint impairment and limits the development of rehabilitation strategies [40]. Limitations of this pilot study are the relatively small sample size (only 140 subjects) and the fact it is not a multicenter trial. In addition, owing to the limited numbers of subjects, we will not compare the efficacy of ITCRP with a regular tai chi program or any other exercise.

In conclusion, this study aims to develop an ITCRP for patients with knee OA, and to estimate the effect of ITCRP intervention on outcomes including pain, function, balance, and joint biomechanics in knee OA. The results of the proposed study will provide a good foundation from which to develop new biomechanics-based exercise therapies for patients with knee OA. Based on the results of the proposed project, further comprehensive research on exercise rehabilitation of knee OA will be proposed.

## Trial status

Patient recruitment.

## Abbreviations

EMG: Electromyography; ITCRP: Innovative tai chi rehabilitation program; OA: Osteoarthritis.

## Competing interests

The authors declare that they have no competing interests.

## Authors’ contributions

YL and J-XL contributed to the conception and design of the trial, and drafting the manuscript. L-YH and X-QW participated in the trial register, communication, and monitoring. X-QW and H-PL participated in the design of statistical analysis. YL, J-XL, X-QW, L-YH, and LW gave final approval of the version to be published. All authors read and approved the final manuscript.
